# Modulation of the S/HgCl_2_ Ratio for the Synthesis and Conversion of Cinnabar and Metacinnabar

**DOI:** 10.3390/nano15030234

**Published:** 2025-02-02

**Authors:** Qilong Hao, Zhehan Zhang, Wenyuan Zhang, Zongren Yu, Yanping Shi, Haixia Zhang, Bomin Su

**Affiliations:** 1 State Key Laboratory of Applied Organic Chemistry, College of Chemistry and Chemical Engineering, Lanzhou University, Lanzhou 730000, China; haoql2023@lzu.edu.cn (Q.H.);; 2 CAS Key Laboratory of Chemistry of Northwestern Plant Resources, Key Laboratory for Natural Medicines of Gansu Province, Lanzhou Institute of Chemical Physics, Chinese Academy of Sciences (CAS), Lanzhou 730000, China; 3 University of Chinese Academy of Sciences, Beijing 100049, China; 4 Gansu Provincial Research Center for Conservation of Dunhuang Cultural Heritage, Dunhuang Academy, Dunhuang 736200, China

**Keywords:** cinnabar, metacinnabar, nanostructures, XRD, SEM, XPS, ICP-MS

## Abstract

Cinnabar has been used as a red pigment for centuries, but its degradation significantly impacts the aesthetic quality of historical paintings, particularly murals. Therefore, investigating the preparation method and transformation process of HgS is highly significant for mural research. In this study, we compared different sulfur sources for HgS synthesis and precisely synthesized α-HgS and β-HgS by adjusting the S/HgCl_2_ ratio. SEM and XRD analyses under optimal conditions demonstrated that spherical β-HgS-1.2 exhibited significant morphological differences in comparison with α-HgS-1.0 and α-HgS-1.5. Elemental analysis of HgS was conducted using XPS and ICP-MS for qualitative and quantitative insights. Based on the potential mechanism of cinnabar discoloration, two strategies for converting black β-HgS to α-HgS were proposed and successfully implemented by adding sulfur or HgCl_2_.

## 1. Introduction

Mercuric sulfide exists in two stable forms: the cubic-phase metacinnabar (β-HgS), which is black, and the hexagonal-phase cinnabar (α-HgS), which is bright red [1]. Cinnabar has played a crucial role as a pigment in Roman wall decoration and has also been utilized in cosmetics and traditional medicine [2,3,4,5,6,7]. In Europe and the Mediterranean region, ancient cinnabar mines are relatively well-documented, and it is feasible to determine the provenances of vermilion by taking into account the isotopes [8]. Cinnabar exists in two forms: natural cinnabar and artificial cinnabar, known as vermilion. Natural cinnabar is formed through near-surface volcanic activity, whereas artificial cinnabar is produced through two main methods: the dry process and the wet process [9]. There are differences in color and stability between natural and artificial cinnabar. Color quality and stability depend on impurities, as in natural cinnabar, or on physical factors in the production process, such as specific gravity, atmospheric conditions, and reaction duration [10]. The stability of natural cinnabar is linked to its elemental composition and geological origin, whereas the stability of artificial cinnabar is influenced by its preparation process and impurities [11]. Owing to the high toxicity of mercury, research on the synthesis of these materials is severely restricted. The majority of the established methods for cinnabar production involve solvothermal techniques, microwave approaches, and phase-transfer methods [12,13,14]. Solvothermal and microwave methods typically necessitate elevated temperatures, while phase-transfer methods are more intricate [15]. High-temperature methods may induce structural defects, whereas room-temperature methods pose challenges in morphology control, frequently leading to the formation of irregularly shaped large particles [16]. Therefore, employing a relatively gentle approach is imperative in synthesizing the two crystal forms of mercury sulfide.

The red pigment α-HgS is known to alter, producing gray, gray-white, or black coloration on the surfaces of degraded artworks [17,18,19]. This phenomenon can significantly impact the aesthetic value of artworks. Therefore, safeguarding this valuable and irreplaceable heritage requires a comprehensive understanding of the degradation mechanisms of pigments. Previous investigations revealed that certain cinnabar samples exhibit a tendency to darken upon exposure to light and halides, forming various compounds [20,21,22,23]. Pliny noted that cinnabar was photosensitive, and its light-induced blackening has been observed throughout history [24,25,26]. The discoloration has been explained as a phase transition to black metacinnabarite by the oxidation of sulfur or by other chemical reactions [27]. Chloride ions act as catalysts in the photoelectrochemical process. Cinnabar then readily undergoes structural transformation, turning black due to the formation of free mercury (Hg^0^) and sulfur (S^0^) in the presence of accumulated chlorides [20]. Factors such as impurities, humidity, and air exposure play a critical role [27,28,29,30]. The sodium chloride in the dirt particles on paints could trigger the same reaction that, in nature, induces the vermilion → corderoite transformation. This could lead to the formation of calomel + S + black HgS upon light exposure. Also, painting technique is a crucial factor in the pigment’s discoloration. The alteration is more pronounced when cinnabar was used alone and absent or reduced when applied together with red lake or red lead [31]. Despite extensive experimental investigations, the complex physicochemical processes underlying cinnabar degradation remain poorly understood [32,33,34].

Metacinnabar, the cubic form of mercury sulfide, which is black, was long believed to be the cause of the dark hue of degraded α-HgS [35,36]. While the transition from α-HgS to β-HgS is complex and lengthy, with many proposed mechanisms, few studies have investigated the conversion of β-HgS to α-HgS [37]. Studying the reverse conversion of β-HgS to α-HgS could facilitate color recovery, providing important insights into the color degradation mechanism of cinnabar.

This study proposed a method for the precise synthesis of α-HgS and β-HgS under relatively mild reaction conditions by controlling the ratio of sulfur to HgCl_2_ and investigated the transformation process between α-HgS and β-HgS. The synthesized HgS was analyzed using X-ray diffraction (XRD) and scanning electron microscopy (SEM) to elucidate the impact of two distinct sulfur sources on HgS synthesis. The elements in HgS were also qualitatively and quantitatively analyzed using X-ray photoelectron spectroscopy (XPS) and inductively coupled plasma mass spectrometry (ICP-MS). Additionally, the successful conversion of β-HgS to α-HgS was achieved by employing two strategies, i.e., adding sulfur or HgCl_2_, resulting in a color change from black to red. Furthermore, the conversion of β-HgS to α-HgS was successfully achieved through the supplementation of sulfur monomers in mock-up painting sample experiments.

## 2. Materials and Methods

### 2.1. Synthesis of HgS

To examine the impact of different raw materials on HgS synthesis, the experiment utilized HgCl_2_ as the source of mercury and two sulfur sources: elemental sulfur (S) and sodium thiosulfate pentahydrate (Na_2_S_2_O_3_·5H_2_O). Taking S as an example, different proportions of HgS were prepared by adjusting the molar ratio of S to HgCl_2_. A certain amount of S (the molar ratio of S to HgCl_2_ ranged from 0.7 to 1.5) was dissolved in 5 mL of ethylenediamine, and then 0.175 mmol HgCl_2_ was added, heated at 80 °C, and stirred at 210 r/min for 4 h. The resulting products were subjected to centrifugation, washed three times with ethanol and ultrapure water, and subsequently dried under vacuum at 60 °C. The HgS products were labeled as HgS-0.7, HgS-0.8, HgS-0.9, HgS-1.0, HgS-1.1, HgS-1.2, HgS-1.3, HgS-1.4, and HgS-1.5. A similar procedure was followed when using Na_2_S_2_O_3_·5H_2_O as the sulfur source. Special reminder: mercury chloride is toxic. This experiment was carried out under the condition of proper safety protection.

### 2.2. Conversion of β-HgS to α-HgS

Firstly, the prepared β-HgS (HgS-1.2) was re-dispersed in 5 mL of ethylenediamine, and 0.053 mmol of sulfur was added. The mixture was then heated and stirred at 80 °C for 2 h to obtain the transformed bright red α-HgS. The successful transformation of β-HgS was confirmed through SEM and XRD analysis.

Alternatively, the prepared β-HgS was re-dispersed in 5 mL of ethylenediamine, and 0.057 mmol of HgCl_2_ was added. The mixture was then heated and stirred at 85 °C for 15 h.

### 2.3. Conversion of α-HgS to β-HgS

The HgS-1.0 sample was redispersed in 5 mL of ethylenediamine, and then 0.034 mmol of sulfur was added. The mixture was heated and stirred at 80 °C for 48 h to investigate the conversion of α-HgS to β-HgS. Similarly, the prepared HgS-1.5 was redispersed in 5 mL of ethylenediamine, and 0.044 mmol of HgCl_2_ was added, with other conditions remaining unchanged.

### 2.4. Mock-Up Painting Samples

To verify the conversion effect of β-HgS to α-HgS in traditional Chinese murals, conversion experiments were conducted using mock-up painting samples, which were prepared with reference to previous literature and slightly modified [38]. A wooden baseboard (1.5 × 1.5 cm^2^) was used as the mock-up painting wall and subsequently coated with calcium sulfate to create the ground layer. After drying naturally, the surface was sanded and smoothed, and a β-HgS pigment mixed with gum arabic was uniformly applied to a thickness of about 0.1–0.2 mm. After drying naturally, it was used as mock-up painting samples.

In order to more accurately replicate the traditional Chinese painting method of mural painting, we also used lime-base simulation samples for conversion experiments. After drying naturally, the surface was sanded and smoothed, and a β-HgS pigment mixed with gum arabic was uniformly applied to a thickness of about 0.1–0.2 mm.

The hydrogels were cut to the size of the mock-up painting samples and moistened with an 18 mM ethylenediamine solution containing a sulfur monomer. Then, the hydrogel was applied to the pigment surface and incubated at 60 °C for 3 h to complete the conversion of β-HgS to α-HgS.

### 2.5. Analytical Techniques

The X-ray diffraction patterns were recorded on an Ultima IV diffractometer (Rigaku, Tokyo, Japan) using CuKα radiation (λ= 1.5418 Å). The scanning electron microscope images were determined on an Apreo S scanning electron microscope (ThermoFisher, Norristown, PA, USA) at 30.0 kV. X-ray photoelectron spectroscopy was used to analyze the surface elemental composition by using Al Kα radiation (Axis Supra XPS system, Shimadzu, Kyoto, Japan). Total Hg measurement was performed on an inductively coupled plasma mass spectrometry (ICP-MS) Plasma Quant PQ9000 (Analytik Jena AG, Jena, Germany).

## 3. Results and Discussion

The selection of raw materials and solvents is crucial for achieving distinct crystal forms, regular morphology, and controlled particle size in HgS synthesis. Several authors have conducted significant research in this area (see Table 1). Typically, four types of Hg and S sources and three solvents have been employed. The use of water as a solvent renders the morphology challenging to control, although the HgS crystal shape can be manipulated by adjusting the synthesis temperature [39]. Solid-state synthesis also does not afford control over the HgS morphology [1]. The coordination effect of the solvent significantly influences crystal morphology. Ethylenediamine and triethylene tetramine, as strong chelating ligands, can create a stable microcrystalline growth environment, leading to the regular morphology of HgS [12,39]. Recognizing the significant impact of particle size and morphology on nanomaterial properties, we successfully synthesized α-HgS using Hg(NO_3_)_2_·H_2_O and HgCl_2_ as raw materials in different solvents. In this study, HgCl_2_ was primarily employed as the starting material for HgS synthesis, and we investigated the influence of temperature and reaction time on the morphology and crystal form of HgS.

### 3.1. Sulfur Source Selection

#### 3.1.1. Na_2_S_2_O_3_ as the Sulfur Source

**Figure 1:**
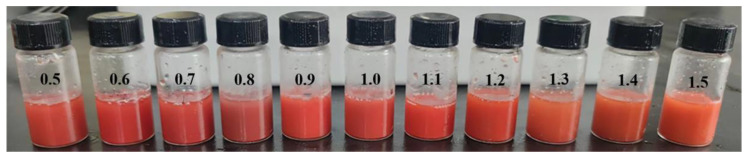
Picture of HgS synthesized with different proportions of Na_2_S_2_O_3_ (Na_2_S_2_O_3_/HgCl_2_ from 0.5 to 1.5).

Initially, Na_2_S_2_O_3_ served as the sulfur source, and the Na_2_S_2_O_3_/HgCl_2_ ratio varied from 0.5 to 1.5 to examine its impact on HgS synthesis. Figure 1 illustrates that the resulting HgS samples exhibited a red color, with XRD analysis confirming the diffraction peaks corresponding to α-HgS (Figure S1). Despite further adjustments to the Na_2_S_2_O_3_/HgCl_2_ ratio, the formation of black β-HgS was not observed, indicating a preference for the synthesis of α-HgS when using Na_2_S_2_O_3_.

#### 3.1.2. Elemental S as the Sulfur Source

**Figure 2:**
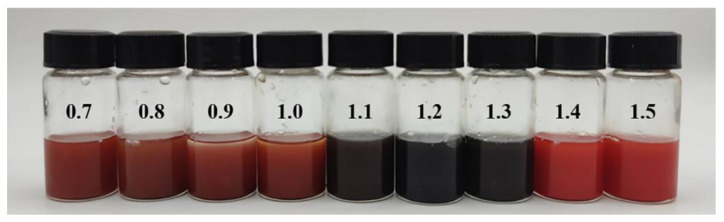
Picture of HgS synthesized with different proportions of S (S/HgCl_2_ from 0.7 to 1.5).

Additionally, the color of HgS exhibits a discernible gradient change when the proportion of sulfur is varied as a raw material. Figure 2 illustrates the successful synthesis of black HgS when the S/HgCl_2_ ratio ranged from 1.1 to 1.3.

The above experiments lead to the conclusion that the choice of sulfur sources significantly influences the synthesis of HgS. Na_2_S_2_O_3_, when used as a sulfur source, predominantly leads to the synthesis of α-HgS, while adjusting the proportion of S allows for the synthesis of HgS with varying colors. Consequently, in the subsequent experiment, S was utilized as the sulfur source, and HgCl_2_ served as the mercury source to further explore the synthesis process.

### 3.2. Characterization of HgS from Different Ratios of S/HgCl_2_

To investigate how varying proportions of sulfur sources affect the formation of α-HgS and β-HgS, SEM analyses were conducted on HgS samples with proportions ranging from 1.0 to 1.5.

**Figure 3:**
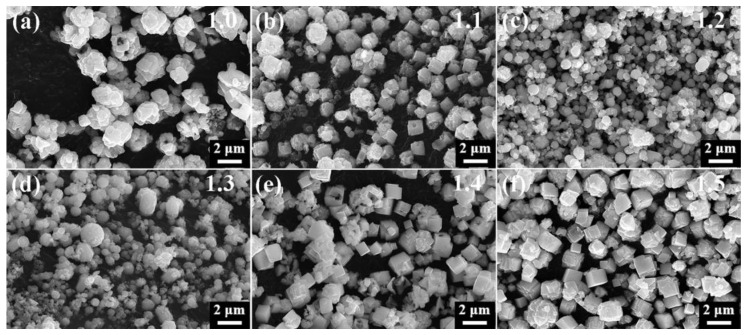
SEM images of HgS with a ratio from 1.0 to 1.5. (**a**) 1.0; (**b**) 1.1; (**c**) 1.2; (**d**) 1.3; (**e**) 1.4; (**f**) 1.5. Experimental conditions shown in Section 2.1.

The SEM images depicted in Figure 3a show the formation of HgS-1.0 as irregular polyhedral agglomerates with a diameter of approximately 1.3 μm. Upon increasing the sulfur proportion to 1.1 (Figure 3b), the irregular polyhedra gradually transitioned into angular shapes, and some polyhedra exhibited a hexahedral stack formation. Subsequently, at a S/HgCl_2_ ratio of 1.2, black β-HgS completely formed, showing a significant morphological change from polyhedral aggregation to a ball-shaped structure with an average particle size of 650 nm, featuring a smooth and evenly dispersed surface (Figure 3c). Further increase in the sulfur proportion to 1.4 resulted in the formation of regular hexahedrons sized approximately 800–1100 nm, along with the transformation of irregular aggregates into small hexahedron stacks (Figure 3e). HgS-1.5 exhibited no significant difference from HgS-1.4, except for a higher abundance and more uniform dispersion of α-HgS regular hexahedrons (Figure 3f).

**Figure 4:**
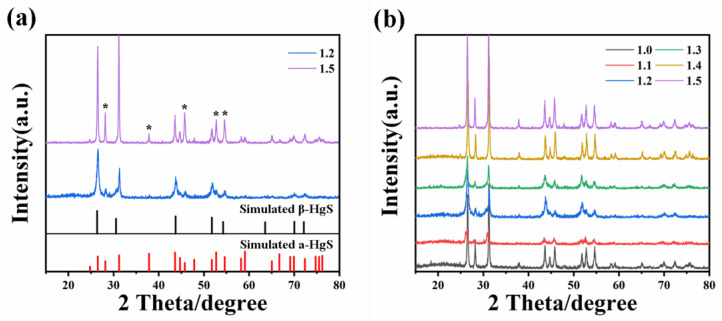
(**a**) Comparison of prepared α-HgS and β-HgS with XRD standard cards; (**b**) XRD plots of different ratios of HgS. The asterisks are used to highlight the peaks.

The experiments demonstrated the controlled synthesis of α-HgS and β-HgS by modulating the S/HgCl_2_ ratio. XRD analysis of different HgS proportions, as depicted in Figure 4a, revealed a high consistency between HgS-1.5 with the XRD standard peaks of cinnabar, and between HgS-1.2 and the XRD standard peaks of metacinnabar, confirming the successful synthesis of α-HgS and β-HgS. In Figure 4b, the diffraction peaks of HgS-1.0 were consistent with those of HgS-1.4 and HgS-1.5, albeit with slightly weakened intensities, indicating that these three proportions represent α-HgS. In comparison to α-HgS, the diffraction peaks of β-HgS-1.2 disappear at 28°, 37°, 45°, and 52°, and the diffraction peaks at 46°, and 54° are noticeably attenuated, whereas HgS-1.1 and 1.3 exhibit a mixed state comprising two different crystal HgS.

**Figure 5:**
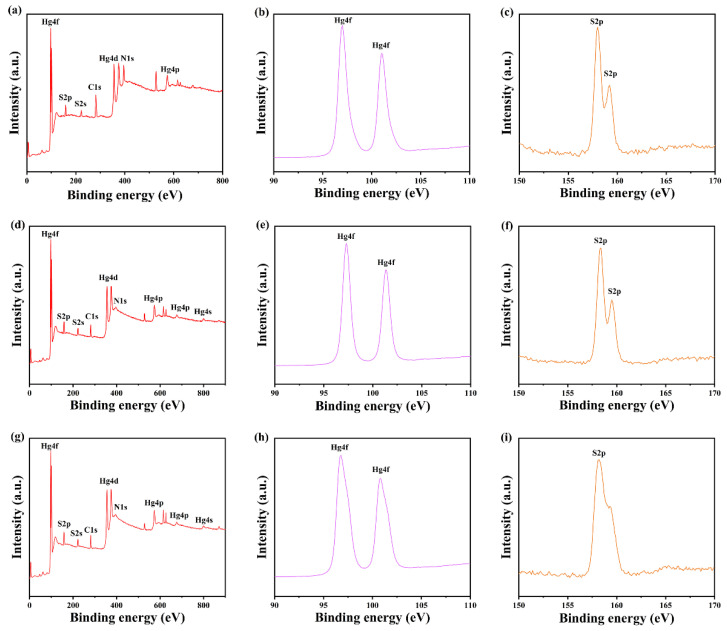
(**a**–**c**) are the XPS profiles of α-HgS-1.0 particles (**a**), Hg4f (**b**), and S2p (**c**), respectively; (**d**–**f**) are the XPS profiles of α-HgS-1.5 particles (**d**), Hg4f (**e**), and S2p (**f**), respectively; (**g**–**i**) are the XPS of β-HgS-1.2 particles (**g**), Hg4f (**h**), and S2p (**i**), respectively.

To investigate the elemental composition of HgS particles and the content of S and Hg elements, α-HgS and β-HgS samples were subjected to XPS and ICP-MS analyses. Figure 5a,d and g display the XPS spectra of α-HgS-1.0, α-HgS-1.5, and β-HgS-1.2 particles, respectively. The XPS spectra reveal Hg, S, N, and C photoelectron peaks within the 0–800 eV range, suggesting high sample purity. In Figure 5b,c the Hg4f and S2p peak area ratio in α-HgS-1.0 particles indicates an S/Hg atomic ratio of approximately 48:52, close to 1:1. The higher surface Hg content may result from Hg^2+^ adsorption and may not accurately represent the overall S/Hg ratio in the sample. Consequently, aqua regia ablation was performed for ICP-MS analysis to obtain the relative S/Hg ratio in the whole sample. The measured S and Hg concentrations in the α-HgS-1.0, α-HgS-1.5, and β-HgS-1.2 samples are presented in Table S1, along with the calculated S/Hg molar ratios of 1.0791, 1.5870, and 1.2513, respectively. These ratios align with the S/HgCl_2_ addition ratios, demonstrating the effectiveness of S/HgCl_2_ ratio modulation in the preparation of α-HgS and β-HgS.

### 3.3. Optimization of Preparation Conditions of HgS

The synthesis conditions for β-HgS-1.2 and α-HgS-1.5, including temperature and reaction time, were optimized. In Figure S2a,b, temperatures below 50 °C resulted in an initial grayish-brown color of the solution after mixing, and no significant change was observed within 4 h. Notably, the color transformation of α-HgS and β-HgS occurred at temperatures exceeding 70 °C, and the most pronounced effect was observed at 80 °C.

**Figure 6:**
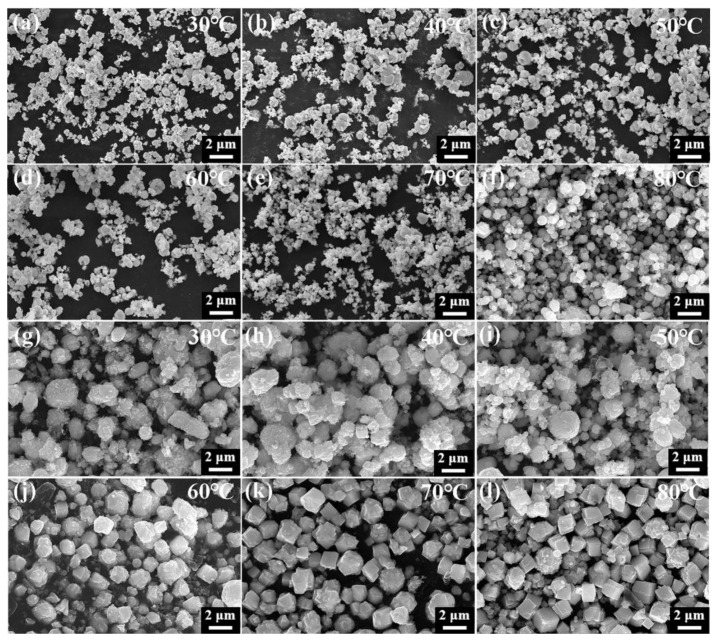
(**a**–**f**): SEM images of HgS-1.2 at different synthesis temperatures from 30 to 80 °C; (**g**–**l**): SEM images of HgS-1.5 at different synthesis temperatures from 30 to 80 °C.

**Figure 7:**
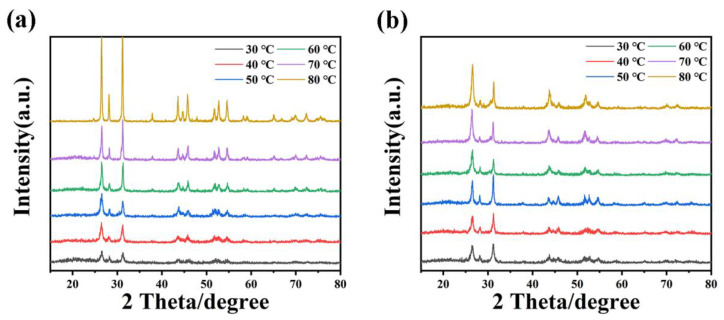
XRD patterns of (**a**) HgS-1.5 and (**b**) HgS-1.2 at different temperatures.

SEM and XRD analyses were conducted of HgS synthesized at various temperatures. Figure 6a–f indicate that with the increase in the reaction temperature, the average particle size of β-HgS-1.2 gradually increases, and uniform spherical particles are formed at 80 °C. The values of the XRD peak height varying with the reaction temperature are shown in Table S2 and Figure 7. As the reaction temperature increases, the height of the diffraction peaks increases, and the highest diffraction peak intensity is presented at 80 °C. Thus, the more uniform the grain size and the more regular the morphology, the higher the intensity of the XRD peaks.

Figure 6g,h depict HgS-1.5 forming 1.5–2.0 μm aggregates at 30–40 °C, with spherical HgS particles appearing 500–600 nm at 50 °C. The similarity between the diffraction peaks of the XRD pattern of HgS at this temperature and those of β-HgS suggests that a small amount of β-HgS may have formed during the synthesis of α-HgS-1.5. As the reaction temperature increased to 60–70 °C, the spherical HgS continued to coordinate with S^2−^, resulting in growth into a polyhedron of approximately 1.2 μm (Figure 6j–k), culminating in the formation of a regular hexahedron morphology at 80 °C (Figure 6l). As shown in Table S3, with the increase in the reaction temperature, the height of the XRD peaks gradually increases. XRD analysis results strongly agreed with SEM observations, indicating that 80 °C favored the growth of HgS particles. Lower temperatures favored β-HgS formation, although incomplete reactions resulted in lower purity. Furthermore, when the reaction temperature exceeded 60 °C, the sulfur proportion primarily regulated HgS morphology. At 80 °C, precise synthesis of α-HgS and β-HgS was possible by controlling the elemental sulfur proportion.

**Figure 8:**
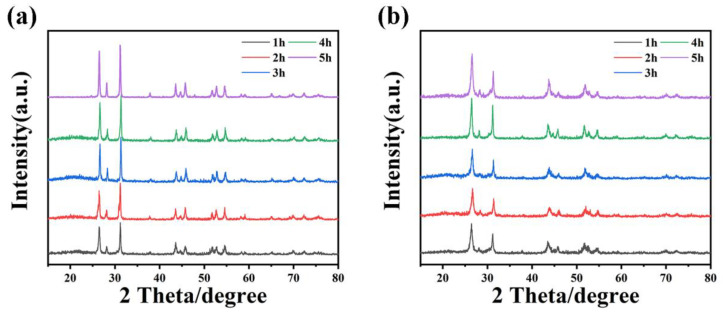
XRD patterns of (**a**) HgS-1.5 and (**b**) HgS-1.2 at different reaction times.

**Figure 9:**
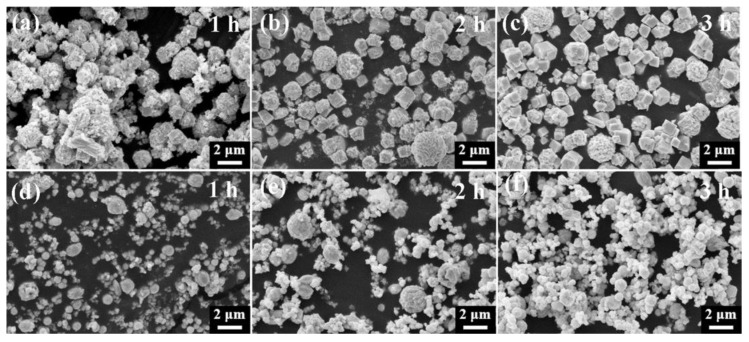
(**a**–**c**): SEM images of the HgS-1.5 reaction from 1h to 3h; (**d**–**f**): HgS-1.2 reaction from 1 h to 3 h.

XRD analyses were conducted on HgS samples at various reaction times, revealing a gradual increase in the intensity of diffraction peaks for α-HgS and β-HgS as reaction time increased, stabilizing after 4 h (Figure 8a,b; Tables S4 and S5). Similarly, SEM images (Figure 9a–c) showed that longer reaction times led to a more regular hexahedral morphology for α-HgS. Additionally, Figure 9d–f illustrated the β-HgS growth process, showing uniform particle size after 3 h. The optimal morphology for both α-HgS and β-HgS was observed after a 4 h reaction, consistent with SEM images and Figure 3c,f. Therefore, the optimal reaction temperature for HgS is 80 °C, with an optimal reaction time of 4 h.

### 3.4. Conversion of β-HgS to α-HgS

Upon completing our experimental investigations, black β-HgS was synthesized at an S/HgCl_2_ ratio of 1.2, whereas α-HgS was obtained at ratios of 1.0 and 1.5. Recent studies employing various spectral techniques have identified calomel and elemental Hg particles in deteriorated segments of wall paintings [28,29]. Several hypotheses propose that mercury loss may contribute to changes in the aesthetic appearance of mural paintings, with the displaced mercury reacting with other substances to form various mercury compounds [39,40,41]. We postulated that changes in the S and Hg ratio within the original α-HgS of the murals over time resulted in the formation of black β-HgS. Given that the synthesis ratio of β-HgS lies between that of α-HgS, two conversion strategies were proposed to facilitate the transformation of β-HgS to α-HgS for the restoration of the mural’s original color: Black β-HgS generation results from the loss of either S or Hg, prompting individual experimental exploration of these two transformation strategies.

#### 3.4.1. Adding S Element for Transformation

In the first transformation strategy, it is hypothesized that prolonged environmental exposure has led to the loss of S from the original α-HgS in the mural painting, resulting in the blackening of α-HgS. Consequently, we conducted the conversion of β-HgS to α-HgS through the supplementary addition of S element to β-HgS, followed by SEM analysis of HgS at various reaction times.

**Figure 10:**
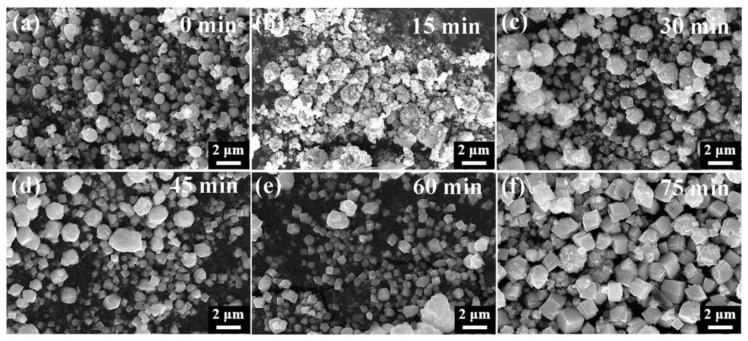
SEM of HgS at different conversion times ((**a**–**f**) conversion time of 0, 15, 30, 45, 60, and 75 min, respectively).

As depicted in Figure 10a, the surface of the spherical β-HgS became rough upon the addition of a specific amount of S, resulting in the transformation of the spherical β-HgS into irregular aggregates composed of small polyhedra after 15 min (Figure 10b). At this stage, the added S re-coordinated with the HgS particles. Following 30 min, the aggregate started to disperse, the smaller polyhedra transformed into a regular hexa-hedral structure, and the solution’s color turned blackish red. After 45 min, the regular hexahedral structure gradually intensified, approaching the morphology of α-HgS (Figure 10d). At 60 min, the spherical β-HgS underwent complete transformation into α-HgS with regular hexahedral morphology (Figure 10e), confirmed by the XRD pattern aligning with the position of the α-HgS diffraction peaks, thereby achieving the conversion from β-HgS to α-HgS (Figure 12a).

#### 3.4.2. Adding Hg Element for Transformation

**Figure 11:**
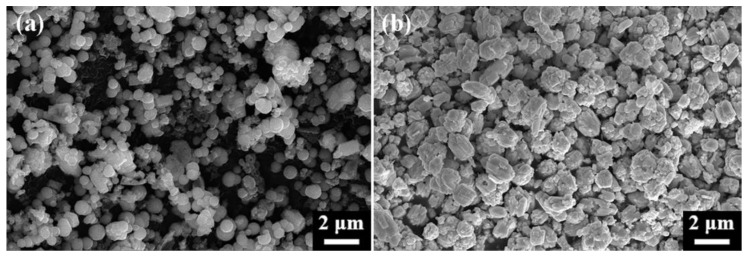
SEM images after adding HgCl₂ at different conversion times: (**a**) 0 h and (**b**) 15 h.

**Figure 12:**
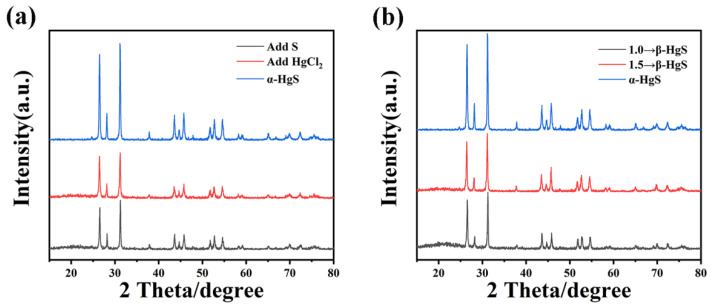
XRD patterns of (**a**) conversion of β-HgS to α-HgS and (**b**) conversion of α-HgS to β-HgS.

In conversion strategy II, we hypothesized that the original red α-HgS turned black due to Hg loss, leading us to convert it to α-HgS-1.0 by adding HgCl_2_. The delayed dissolution of HgCl_2_ in the mixed solution necessitates a longer conversion duration (Figure 11). The XRD pattern confirms that converting β-HgS to α-HgS is possible by adding HgCl_2_, albeit with more difficulty than the first strategy (Figure 12a).

### 3.5. Conversion of α-HgS to β-HgS

**Figure 13:**
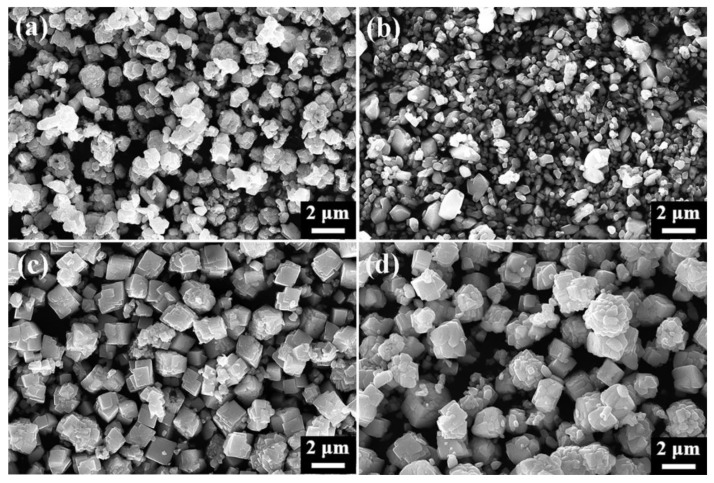
(**a**,**b**) SEM images before and after the conversion from HgS-1.0 to β-HgS, respectively; (**c**,**d**) SEM images before and after the conversion from HgS-1.5 to β-HgS, respectively.

Under the same experimental conditions, an α-HgS to β-HgS conversion experiment was conducted with the addition of elemental S and HgCl_2_. The SEM images in Figure 13a,b show that conversion was incomplete after 48 h of reaction with elemental S. Figure 13c,d show that after 48 h of reaction with added HgCl_2_ to HgS-1.5, the hexahedral morphology of α-HgS remained, with no spherical β-HgS observed. Similarly, the XRD pattern in Figure 12b indicates that the diffraction peak position of α-HgS remained unchanged after 48 h, with only slight peak intensity weakening, likely due to surface roughness from the addition of HgCl_2_ and S. This suggests that α-HgS exhibits greater stability and is less easily converted to β-HgS.

### 3.6. Mock-Up Painting Samples

**Figure 14:**
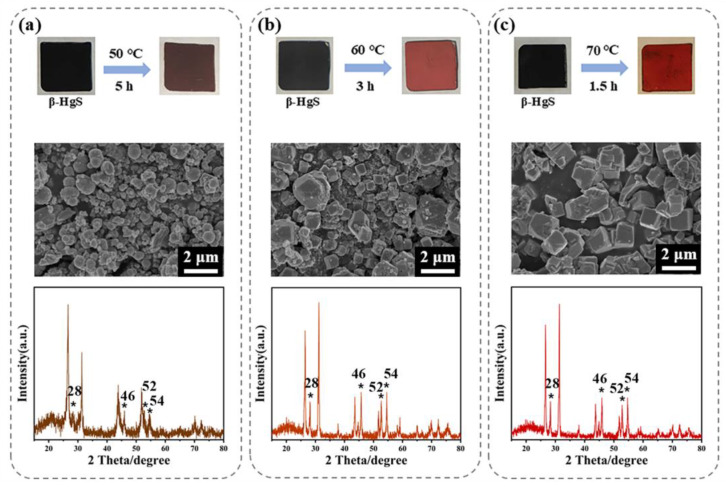
Conversion of β-HgS to α-HgS in mock-up painting samples on calcium sulfate substrates. Color changes and SEM and XRD images of HgS at different transformation temperatures: (**a**) 50 °C; (**b**) 60 °C; (**c**) 70 °C. (The asterisks are used to highlight the four peaks at 28, 46, 52, and 54.)

To verify the conversion of β-HgS to α-HgS, experiments were conducted using mock-up painting samples prepared on two substrates: calcium sulfate and lime. To ensure mild transformation conditions, the experiments were conducted at three temperatures: 50 °C, 60 °C, and 70 °C. The transformation experiment of mock-up painting samples on calcium sulfate is shown in Figure 14. Brown HgS formed at a conversion temperature of 50 °C, with a morphology mainly spherical and heterogeneous in size (Figure 14a), remaining similar to that of β-HgS (Figure 3c). The XRD pattern was consistent with β-HgS (Figure 4a), with only weak α-HgS characteristic peaks appearing at 28° and 46°, indicating that the conversion process was difficult to realize at 50 °C. At 60 °C, as shown in Figure 14b, a large amount of spherical HgS converted to a hexahedral shape, and the diffraction peaks of the XRD pattern at 28°, 46°, 52°, and 54° were significantly enhanced, consistent with α-HgS, proving successful conversion. At 70 °C, it took 1.5 h for the mock-up painting samples to complete conversion from β-HgS to α-HgS, faster than the conversion at 60 °C (3 h). Higher temperatures accelerated the HgS crystallization process by increasing the solute diffusion rate.

The transformation experiments on lime substrate mock-up painting samples are shown in Figure S4. At 50 °C, the SEM image still displays spherical β-HgS, indicating that the transformation process is incomplete. According to the optimization of the synthesis temperature discussed in Section 3.3, the formation of α-HgS requires a temperature higher than 60 °C. As the temperature increases to 60 °C and 70 °C, the spherical β-HgS transforms into hexahedral-shaped α-HgS, with XRD patterns confirming the formation of α-HgS (Figure S4b,c). Consequently, the conversion of β-HgS to α-HgS is achievable at reaction temperatures exceeding 60 °C in the mock-up painting samples.

While the transformation mechanism showed promising results in mock-up painting samples, the complexity of real case studies presents numerous challenges. In traditional mural paintings, cinnabar is embedded in complex matrices, such as calcium carbonate support, and is subject to interference from other pigments and binding media. These matrices can affect the chemical and physical behavior of cinnabar, posing challenges to the proposed conversion mechanisms. Light irradiation can cause cinnabar to undergo an oxidation reaction, exhibiting typical semiconductor properties. Moreover, the results of photochemical reaction kinetics indicate that doping with minium (Pb_3_O_4_) may accelerate the photodegradation process [42]. Halogen impurities, especially chloride ions, play a significant role in the degradation of cinnabar. Mercury chlorides such as HgCl_2_, Hg_2_Cl_2_, and Hg_3_S_2_Cl_2_ have been detected in blackened cinnabar. These are likely intermediate products of cinnabar degradation, demonstrating the chemical reaction between cinnabar and specific salts. Pollutants in the air, such as dust and sulfur dioxide, can adhere to the surface of cinnabar, making its surface become duller or turn gray. In addition, the proposed conversion strategy requires supplementing S or HgCl_2_, and it remains uncertain whether these interventions will affect the original mural system, necessitating further indepth study.

## 4. Conclusions

In summary, regular hexahedral α-HgS and spherical β-HgS were precisely synthesized by adjusting the S/HgCl_2_ ratio and reaction temperature. Black β-HgS was successfully converted to red α-HgS using two conversion strategies in a solution system. The restoration of cinnabar blackening in mock-up painting samples was achieved by supplementing S monomers. While the proposed conversion strategies require introducing cinnabar raw materials and have not yet achieved non-intervention conversion, they are expected to provide valuable reference information for restoring cinnabar/vermilion red patterns in murals. The larger particle size of α-HgS compared to β-HgS may hinder the complete conversion of crystalline α-HgS to β-HgS. We will further investigate the conversion of α-HgS to β-HgS by reducing its particle size through grinding.

## Figures and Tables

**Table 1:** The synthesis scheme of HgS in previous studies.

Previous Studies	Hg Raw Material	S Raw Material	Solvent	Morphology of Hgs
Taigyu Lee [1]	Hg	Sulfur	—	β-HgS
Jinghui Zeng [12]	Hg(NO_3_)_2_·H_2_O	Na_2_S·9H_2_O	Triethylene tetraamine	Rod-like α-HgS
Wenkang Zhang [39]	HgCl_2_	Na_2_S·9H_2_O	Ultrapure water	α-HgS + β-HgS
Jianhui Zhang [40]	HgO	Sulfur	Ethylenediamine	Spheric + Rod-like HgS
Jiangshan Shen [40]	Hg(NO_3_)_2_·H_2_O	Na_2_S_2_O_3_·5H_2_O	Ultrapure water	α-HgS
		Thiourea		β-HgS
This work	HgCl_2_	Sulfur	Ethylenediamine	α-HgS + β-HgS

## Data Availability

The original contributions presented in this study are included in the article/Supplementary Materials.

## Supplementary Materials

The following supporting information can be downloaded at: https://www.mdpi.com/article/10.3390/nano15030234/s1, Figure S1: XRD patterns of HgS prepared with Na2S2O3 and HgCl2; Figure S2: Reaction temperature from 30 to 80 °C HgS color change ((a)β-HgS-1.2 and (b)α-HgS-1.5); Figure S3: SEM images of HgS with a S/HgCl2 ratio from 0.7 to 0.9; Figure S4: Conversion of β-HgS to α-HgS in mock-up painting samples on lime substrates. Color changes, SEM and XRD images of HgS at different transformation temperatures: (a) 50 °C; (b) 60 °C; (c) 70 °C; Table S1: ICP-MS analysis of α-HgS-1.0, α-HgS-1.5 and β-HgS-1.2; Table S2: XRD diffraction peak heights of β-HgS-1.2 at different reaction temperatures; Table S3: XRD diffraction peak heights of α-HgS-1.5 at different reaction temperatures; Table S4: XRD diffraction peak heights of α-HgS-1.5 at different reaction times; Table S5: XRD diffraction peak heights of β-HgS-1.2 at different reaction times.
