# Supplementary material for: Modulation of the S/HgCl2 Ratio for the Synthesis and Conversion of Cinnabar and Metacinnabar
Source: Nanomaterials (Basel). 2025 Feb 2;15(3):234. doi: 10.3390/nano15030234 (PMC11819968; doi:10.3390/nano15030234)
Supplement: Supplementary file 1 [file nanomaterials-15-00234-s001.zip › nanomaterials-3441925-supplementary.pdf]

Supporting Information

Modulation of the $S/HgCl_2$ ratio for the synthesis and conversion of cinnabar and metacinnabar

Qilong Hao [1,2,3], Zhehan Zhang [1], Wenyuan Zhang [4], Zongren Yu [4], Yanping Shi [2], Haixia Zhang[1*], Bomin Su [4]

[1] *State Key Laboratory of Applied Organic Chemistry, College of Chemistry and Chemical Engineering, Lanzhou University, Lanzhou 730000, China*

[2] *CAS Key Laboratory of Chemistry of Northwestern Plant Resources, Key Laboratory for Natural Medicines of Gansu Province, Lanzhou Institute of Chemical Physics, Chinese Academy of Sciences (CAS), Lanzhou 730000, China*

[3] *University of Chinese Academy of Sciences, Beijing 100049, China*

[4] *Gansu Provincial Research Center for Conservation of Dunhuang Cultural Heritage, Dunhuang Academy, 736200 Dunhuang, China*

**Corresponding author: * Haixia Zhang. e-mail address: zhanghx@lzu.edu.cn.*

Appendix: Experimental Section

Chemicals.

All chemicals and reagents used were at least of analytical grade. Mercury chloride ($HgCl_2$) was purchased from Guizhou Tongren Yinhu Chemical Co. Ltd (Guizhou, China). Sulfur and sodium thiosulfate pentahydrate ($Na_2S_2O_3 \cdot 5H_2O$) were purchased from Shanghai Chemical Reagent Factory (Shanghai, China). Ethanediamine (99%) and ethanol (95%) were supplied from Alfa Aesar Company (Tianjin, China).

Instrumentations.

The X-ray diffraction patterns were recorded on an Ultima IV diffractometer (Rigaku, Japan) using CuKα radiation (λ= 1.5418 Å). The scanning electron microscope images were determined on an Apreo S scanning electron microscope (ThermoFisher, USA) at 30.0 kV. X-Ray photoelectron spectroscopy was used to analyze the surface elemental composition by using Al Kα radiation (Axis Supra XPS system, Shimadzu, JPN). Total Hg measurement was performed on an Inductively coupled plasma-Mass Spectrometry (ICP-MS) Plasma Quant PQ9000 (Analytik Jena AG, Germany).

Appendix: Results and discussion

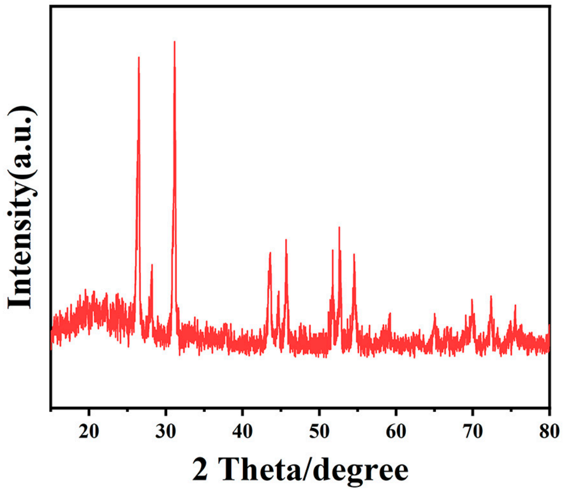

Figure S1. XRD patterns of HgS prepared with $Na_2S_2O_3$ and $HgCl_2$.

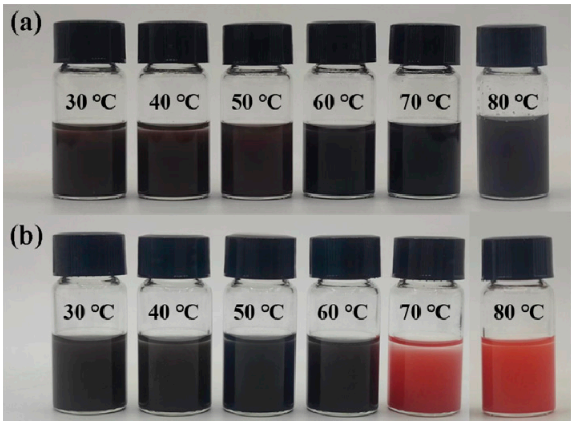

Figure S2. Reaction temperature from 30 to 80 °C HgS color change ((a)β-HgS-1.2 and (b)α-HgS-1.5).

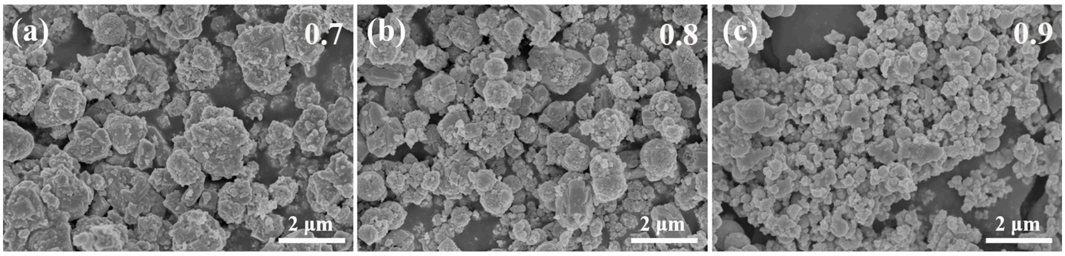

Figure S3. SEM images of HgS with a S/$HgCl_2$ ratio from 0.7 to 0.9.

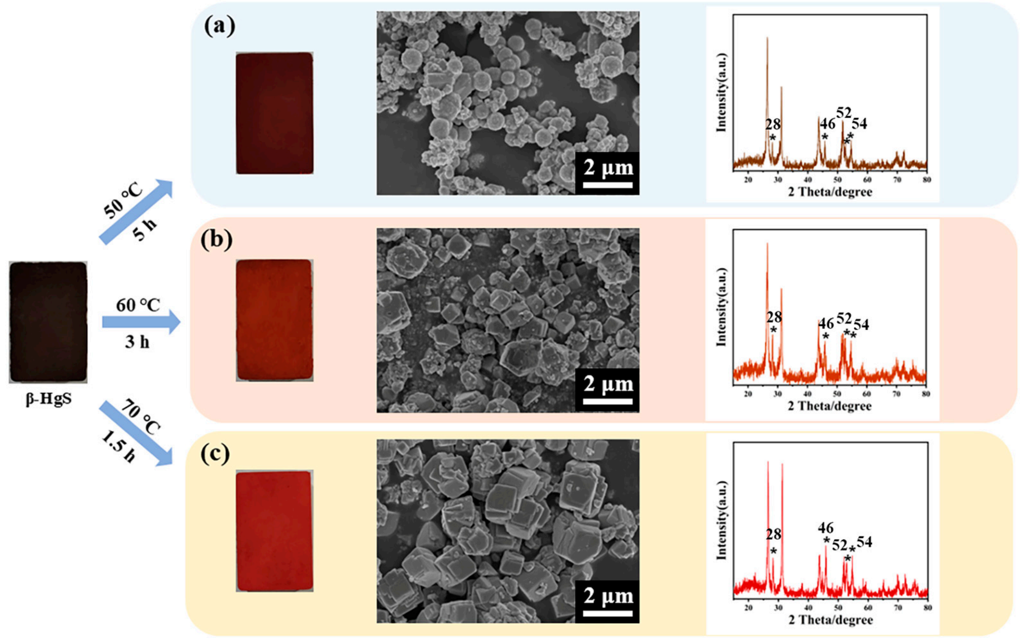

Figure S4. Conversion of β-HgS to α-HgS in mock-up painting samples on lime substrates. Color changes, SEM and XRD images of HgS at different transformation temperatures: (a) 50°C; (b) 60 °C; (c) 70 °C.

Table S1. ICP-MS analysis of α-HgS-1.0, α-HgS-1.5 and β-HgS-1.2.

		Conc.1(mg/L)	Conc.2(mg/L)	S/Hg(molar ratio)
α-HgS-1.0	Hg	0.1767	0.1793	1.0791
	S	0.0309	0.0305	
α-HgS-1.5	Hg	0.1680	0.1659	1.5870
	S	0.0424	0.0423	
β-HgS-1.2	Hg	0.1909	0.1921	1.2513
	S	0.0388	0.0378	

Table S2. XRD diffraction peak heights of β-HgS-1.2 at different reaction temperatures.

β-HgS-1.2 (reaction temperature)						
	Height					
2-Theta	30°C	40°C	50°C	60°C	70°C	80°C
26.48	386	457	556	551	834	1054
31.20	369	460	682	366	519	531
43.72	130	182	163	230	332	407
51.82	116	108	159	158	314	324

Table S3. XRD diffraction peak heights of α-HgS-1.5 at different reaction temperatures.

α-HgS-1.5 (reaction temperature)						
	Height					
2-Theta	30°C	40°C	50°C	60°C	70°C	80°C
26.50	327	450	578	658	788	1557
28.16	144	92	189	178	198	603
31.14	198	370	341	374	563	1633
43.62	93	182	197	227	313	638
45.74	61	196	142	154	336	689
51.76	50	190	191	117	247	325
52.74	101	96	133	200	201	528
54.60	95	136	118	123	254	533

Table S4. XRD diffraction peak heights of α-HgS-1.5 at different reaction times.

α-HgS-1.5 (reaction time)					
	Height				
2-Theta	1h	2h	3h	4h	5h
26.50	681	679	767	798	817
28.16	206	261	292	278	394
31.14	629	836	1222	1028	1401
43.62	275	252	237	220	256
45.74	247	269	267	257	336
51.76	200	208	182	118	187
52.74	205	230	310	214	304
54.60	231	250	271	298	276

Table S5. XRD diffraction peak heights of β-HgS-1.2 at different reaction times.

β-HgS-1.2 (reaction time)					
	Height				
2-Theta	1h	2h	3h	4h	5h
26.48	663	513	515	957	941
31.20	440	467	487	776	650
43.72	265	230	262	347	407
51.82	190	206	290	316	324
